# Phosphomimetic cardiac myosin-binding protein C partially rescues a cardiomyopathy phenotype in murine engineered heart tissue

**DOI:** 10.1038/s41598-019-54665-2

**Published:** 2019-12-03

**Authors:** Alexander Dutsch, Paul J. M. Wijnker, Saskia Schlossarek, Felix W. Friedrich, Elisabeth Krämer, Ingke Braren, Marc N. Hirt, David Brenière-Letuffe, Alexandra Rhoden, Ingra Mannhardt, Thomas Eschenhagen, Lucie Carrier, Giulia Mearini

**Affiliations:** 1 Institute of Experimental Pharmacology and Toxicology, University Medical Center Hamburg-Eppendorf, Hamburg, Germany; 2 DZHK (German Centre for Cardiovascular Research), partner site Hamburg/Kiel/Lübeck, Hamburg, Germany; 3 Vector Core Unit, Institute of Experimental Pharmacology and Toxicology, University Medical Center Hamburg-Eppendorf, Hamburg, Germany; 4 Present Address: Technical University Munich, School of Medicine, German Heart Centre, Department of Cardiology, Munich, Germany; 5 Present Address: Department of Physiology, Amsterdam Cardiovascular Sciences, Amsterdam UMC, location VUmc, Amsterdam, The Netherlands

**Keywords:** Cardiac hypertrophy, Gene therapy

## Abstract

Phosphorylation of cardiac myosin-binding protein C (cMyBP-C), encoded by *MYBPC3*, increases the availability of myosin heads for interaction with actin thus enhancing contraction. cMyBP-C phosphorylation level is lower in septal myectomies of patients with hypertrophic cardiomyopathy (HCM) than in non-failing hearts. Here we compared the effect of phosphomimetic (D282) and wild-type (S282) cMyBP-C gene transfer on the HCM phenotype of engineered heart tissues (EHTs) generated from a mouse model carrying a *Mybpc3* mutation (KI). KI EHTs showed lower levels of mutant *Mybpc3* mRNA and protein, and altered gene expression compared with wild-type (WT) EHTs. Furthermore, KI EHTs exhibited faster spontaneous contractions and higher maximal force and sensitivity to external [Ca^2+^] under pacing. Adeno-associated virus-mediated gene transfer of D282 and S282 similarly restored *Mybpc3* mRNA and protein levels and suppressed mutant *Mybpc3* transcripts. Moreover, both exogenous cMyBP-C proteins were properly incorporated in the sarcomere. KI EHTs hypercontractility was similarly prevented by both treatments, but S282 had a stronger effect than D282 to normalize the force-Ca^2+^-relationship and the expression of dysregulated genes. These findings in an *in vitro* model indicate that S282 is a better choice than D282 to restore the HCM EHT phenotype. To which extent the results apply to human HCM remains to be seen.

## Introduction

Hypertrophic cardiomyopathy (HCM) is the most common inherited cardiac disease with an estimated prevalence in the general population of 0.2%^1^. After more than 20 years, Semsarian *et al*. proposed to revise the estimated prevalence to more than 1 in 500 people, since advances in cardiovascular medicine, such as diagnostic cardiac imaging and genetic testing have been made^2^. Principal hallmarks of HCM are left ventricular hypertrophy, diastolic dysfunction, myocardial/myofibrillar disarray and interstitial fibrosis. It is clinically characterized by an unexplained increase of the left-ventricular wall thickness (≥15 mm in adults) and an asymmetric hypertrophy of the left ventricle (LV) most commonly including the interventricular septum^3^. Diastolic dysfunction appears relatively early, whereas the systolic function is affected only later during the disease progression^4^. Besides the transition to heart failure, HCM can result in sudden cardiac death, the latter occurring especially in young competing athletes^5^. HCM is an autosomal-dominant inherited cardiac disease and it may be considered a sarcomeropathy, since it is associated to several variants mainly in sarcomeric genes. The most frequently affected gene is *MYBPC3*, encoding cardiac myosin-binding protein C (cMyBP-C)^6^. Noteworthy, about 60% of the known *MYBPC3* variants are nonsense or frameshift^6,7^, resulting in C-terminally truncated proteins^8,9^. However, these truncated proteins remain undetectable in septal myectomies from HCM patients^10,11^. Involvement of the nonsense-mediated mRNA decay as well as of the ubiquitin-proteasome system and the autophagy-lysosomal pathway has been shown in the development of HCM in murine models and in patients^12–15^. Absence of cMyBP-C disturbs the stoichiometry of the sarcomere, impairing its function and particularly relaxation, suggesting haploinsufficiency as the main disease mechanism^10,16^. Nevertheless, the presence of poison polypeptides that result from different mutant *MYBPC3* mRNAs may be involved in disease pathogenesis. Therefore, lower amount of cMyBP-C (=haploinsufficiency) and the presence of mutant cMyBPC, which could interfere with sarcomere function or other cellular mechanisms (=poison polypeptide) are non-exclusive pathomechanisms that probably depend on the nature of *MYBPC3* mutation occurring in HCM patients^17^.

cMyBP-C is a sarcomeric protein composed of 8 immunoglobulin-like and 3 fibronectin domains. Unique to the cardiac isoform are the C0 domain, a Pro-Ala rich linker region between the C0 and C1 domains, and a regulatory motif (M-motif) between C1 and C2 domains carrying 4 phosphorylation sites. Phosphorylation of cMyBP-C regulates the interaction of thick and thin filaments by abolishing its binding to myosin-S2 and allowing strong interaction of myosin heads with actin filaments. This post-translational modification of cMyBP-C has been shown to be essential for maintaining a normal cardiac function even at rest^8,18–20^, to be cardioprotective^21^ and to shield the protein itself from degradation, which might preserve cardiac contractility^19^. Among the four phosphorylatable serine residues within the M-Motif (murine Ser-273, Ser-282, Ser-302 and Ser-307), Ser-282 is the first target of protein kinases after β-adrenergic stimulation and might modulate phosphorylation of the remaining serine residues in a hierarchical manner^22,23^. Our group previously developed a homozygous *Mybpc3*-targeted knock-in (KI) mouse carrying a founder truncating *MYBPC3* mutation, which accounts for 14% of HCM cases in Tuscany, Italy^24^. In KI mice total *Mybpc3* mRNA level is 80% lower than in wild-type (WT) littermates resulting in only 10% of protein compared to WT^15^. Interestingly, the point mutation results in different mutant *Mybpc3* mRNAs. In homozygous KI mice *Mybpc3* gene therapy enabled long-term disease prevention improving cardiac function and correcting both haploinsufficiency and poison peptide pathomechanisms^25^. At the same time it has been shown that in engineered heart tissues (EHTs), three-dimensional heart muscle strips^26,27^ generated from *Mybpc3* KI cardiac cells, *Mybpc3* gene transfer prevented the development of hypercontractility and accelerated kinetics exhibited by KI EHTs^25,28^.

The goal of the present study was to investigate whether and to which extent cMyBP-C carrying a charged aminoacid (aspartic acid) at position 282 (D282) and thus mimicking permanent phosphorylation is able to prevent the HCM phenotype in KI EHTs compared to wild-type cMyBP-C with phosphorylatable serine at that position (S282). Therefore, KI EHTs were transduced with adeno-associated virus serotype 6 (AAV6), encoding either D282 or S282 cMyBP-C. We performed molecular analyses of *Mybpc3* mRNAs and cMyBP-C protein levels, expression analysis of genes encoding proteins related to hypertrophic signaling, Ca^2+^-, K^+^-, Na^+^-handling, and sarcomere components, as well as measurements of contractile parameters.

## Methods

### Animals

The investigation conforms to the guidelines for the care and use of laboratory animals published by the NIH (Publication No. 85–23, revised 1985). The experimental procedures were in accordance with the German Law for the Protection of Animals and accepted by the Ministry of Science and Public Health of the City State of Hamburg, Germany (ORG612). *Mybpc3*-targeted KI mice were generated previously^15^, and both KI and WT mice were maintained on a Black Swiss genetic background.

### Production and titration of adeno-associated virus particles

The AGT codon corresponding to serine 282 (S282) in the wild-type *Mybpc3* cDNA was mutated to GAT coding for aspartic acid (D282) via site-directed mutagenesis by PCR using the QuikChange II XL Site-Directed Mutagenesis Kit (Agilent Technologies; primers are given in Suppl. Table S1 ) and verified by sequencing. Both constructs, S282 and D282, were FLAG-tagged and under the human cardiac troponin T (*TNNT2*) promoter. AAV6 pseudo-typed vectors were generated by co-transfection of HEK293 cells with the pdsAAV-*TNNT2*-FLAG-*Mybpc3* transfer plasmid (S282 or D282) or pdsAAV-CMV (for “empty” virus) and the AAV-packaging plasmid pDP6rs (kind gift from Juergen Kleinschmidt, DKFZ Heidelberg), which provides the AAV2 rep and AAV6 cap genes and adenoviral helper functions. Generation of recombinant AAV6 particles was performed as described previously^25^.

### Engineered heart tissue generation, transduction and contraction measurements

EHTs were generated from freshly isolated, unpurified cardiac cells from 0- to 1-day-old mice as described before^17,25,28^. Briefly, isolated cells were resuspended at a density of 6.8 × 10^6^ cells/ml in medium containing bovine fibrinogen (5 mg/ml), aprotinin (2.5 μg/ml), 2x DMEM (55 µl/ml) and 10% Matrigel (BD Bioscience). The reconstitution mix (100 μl/EHT) was mixed with bovine thrombin (3 μl/EHT, 100 U/ml, Biopur) and pipetted into the rectangular agarose casting molds in a 24-well plate containing silicon racks with four pairs of elastic posts (EHT Technologies GmbH). After fibrin polymerization (2 h at 37 °C, 7% CO_2_), which led to formation of a muscle strip around the tips of the silicone posts, the racks were transferred to fresh 24-well plates containing culture medium (DMEM, 10% horse serum, 2% chick embryo extract, 1% penicillin/streptomycin, 10 μg/ml insulin and 33 μg/ml aprotinin), and maintained at 21% O_2_, 7% CO_2_ and 37 °C in a humidified (>90%) incubator for 16–21 days. On day 5, cytosine β-D-arabinofuranoside (25 μg/ml) was added to the culture medium for 48 h to prevent proliferation of non-cardiomyocytes. AAV6 transduction (MOI of 1,000 vg/cell) with D282, S282 or empty constructs was performed in freshly isolated neonatal cardiac cells before adding reconstitution mix components and casting EHTs^17,25,29,30^. Spontaneous beating of EHTs was measured over time via automated video-optical recording^26^. In addition, EHTs were electrically stimulated at 6 Hz by mounting the silicone racks onto electrical pacing units (EHT Technologies GmbH) as described previously^17,31^. The signals were generated by a Grass S88X Dual Output Square Stimulator (Natus Neurology Incorporated, Warwick, USA). An output voltage of 2 V (yielding an electrical field strength of 2 V/cm) in biphasic pulses of 4 ms was applied. Measurements were performed in Tyrode’s solution (in mM: NaCl 120, KCl 5.4, MgCl_2_ 1.0, CaCl_2_ 0.1–1.8, NaH_2_PO_4_ 0.4, NaHCO_3_ 22.6, glucose 5.0, Na_2_EDTA 0.05, ascorbic acid 0.3). The external Ca^2+^ concentration [Ca^2+^] was increased stepwise every 10 min and force measured directly before the next step. Contractility measurements were performed on days 14 and 15. Values for average force, contraction and relaxation velocities, contraction time (T1_20%_) and relaxation time (T2_20%_) were calculated with the machine’s respective EHT analysis software (EHT Technologies GmbH).

### RT-PCR and RT-qPCR

Total RNA was extracted from single EHTs with the TRIzol reagent (Invitrogen, 300 μl/EHT) following the manufacturers’ instructions and further processed as described before^17,25^. RT-qPCR was performed using either SYBR Green or TaqMan probes and the TaqMan ABI Prism 7900HT sequence detection system (Applied Biosystems). Messenger RNA levels were measured in triplicates and normalized to *Gnas*. Quantification of mRNA levels was done using the 2^−ΔΔCt^ method and related to WT values. Primers are given in Suppl. Table S2.

### Gene expression analysis

Gene expression analysis was performed with a customized nanoString’s nCounter Element Tag-set panel of 84 genes coding for proteins involved in hypertrophy, Ca^2+^, K^+^ and Na^+^ handling and constituents of the sarcomere or other intermediate filaments. Same quantity of RNAs of three EHTs in each group were pooled, and 50 ng were used for the run on the nCounter Sprint Profiler following manufacturer’s instructions. Raw data were analyzed with the nSolver software using 4 housekeeping genes for normalization (*Abcf1, Cltc, Gapdh* and *Pgk1*). Data were expressed as fold-change over WT-EHT levels, only genes that were lower than 0.7-fold or higher than 1.3-fold expressed in non-transduced KI EHTs were selected. Heatmaps were generated with the freeware Morpheus (https://software.broadinstitute.org/morpheus/). No statistical analysis was performed since n = 1 pool in each group.

### Protein extraction and Western blot analysis

EHTs were detached from the silicone posts, washed two times with PBS and then processed by mechanical tissue lysing with stainless steel beads (Qiagen TissueLyser, 1 min, 25 Hz) in M-PER Mammalian Protein Extraction reagent (Thermo Scientific), supplemented with protease and phosphatase inhibitors cocktails (Roche). After addition of Laemmli-buffer, samples were boiled for 5 min at 95 °C. For Western blot analysis, denatured proteins were separated on 10–12% SDS-polyacrylamide (29:1) mini-gels (Bio-Rad) and electrotransferred onto nitrocellulose membranes. After blocking for 1 h at room temperature in 5% non-fat milk solution in 1x TBS-T, membranes were stained with primary antibodies directed against the FLAG epitope (1:5,000; Sigma #F3165), cMyBP-C (1:1,000; M-motif gift from C. Witt, Heidelberg), α-actinin (1:1,000; Sigma #A7811), pSer-273-cMyBP-C (1:2,000^32^), pSer-282-cMyBP-C (1:1,000; custom-made), pSer-302-cMyBP-C (1:10,000^32^). Secondary antibodies were anti-mouse (1:20,000; Dianova #515-035-003) or anti-rabbit (1:6,000; Sigma #A0545) peroxidase-conjugated. Immunoreactive bands were visualized by ECL-Prime Kit detection (GE Healthcare) and signals were detected with ChemiGenius2 Bio Imaging System. α-Actinin was used as a loading control.

### Immunofluorescence analysis of EHTs

Whole EHTs were washed in PBS and fixed with Roti-Histofix (Carl Roth GmbH, P087) at 4 °C overnight. After removal from the posts, EHTs were incubated in blocking solution at 4 °C overnight (10% FCS, 1% BSA, 0.5% Triton X-100 in PBS). After permeabilization, EHTs were stained at 4 °C overnight in blocking solution without FCS with primary antibodies directed against the FLAG epitope (1:800; Sigma #F3165), cMyBP-C (1:200; M-motif gift from C. Witt, Heidelberg) and α-actinin (1:200; Sigma #A7811). Anti-mouse IgG Alexa 488-conjugated (1:800; Life Technologies #A11029) and anti-rabbit IgG Alexa 546-conjugated (1:800; Life Technologies #A11035) were used as secondary antibodies. Nuclei were stained with DRAQ5 (1:1,000; BioStatus). EHTs were finally mounted with Mowiol-488 between object slide and coverslips. Confocal images were acquired with a Zeiss LSM 710 system.

### Statistical analysis

All data were expressed as mean ± SEM and analyzed with the software GraphPad Prism 8. Statistical analysis comparing the effect of isoprenaline in the same group was done using the paired Student’s t-test. More than two groups were statistically compared by a one-way ANOVA analysis followed by Dunnett’s multiple comparison test. Force-calcium relationship data were analyzed using the Hill equation, with EC_50_ as the free Ca^2+^ concentration, which yields 50% of the maximal force and nH representing the Hill coefficient. Curves were fitted to the data points and force-pCa relationship comparison was done by using extra sum-of-squares F-test^48^. A value of *P* < 0.05 was considered statistically significant.

## Results

### D282 and S282 cMyBP-C gene transfer prevents accumulation of mutant mRNAs and restores protein levels

**Figure 1:**
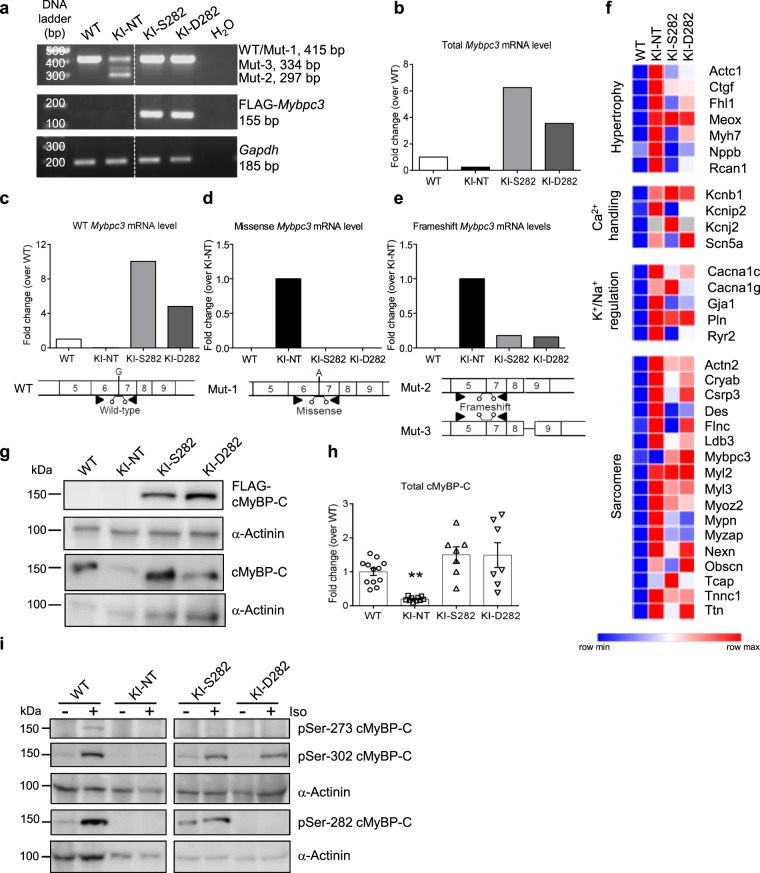
Molecular phenotype on mRNA and protein levels after *Mybpc3* gene transfer. Single wild-type (WT) and knock-in (KI) EHTs either non-transduced (NT) or transduced with AAV6 (MOI 1,000 vg/cell) encoding wild-type cMyBP-C (S282) or phosphomimetic cMyBP-C (D282) were used to extract total RNA and were pooled (200 ng RNA/sample; 3 EHTs per group) prior to reverse transcription. (**a**) Representative agarose gel of RT-PCR with different primer pairs. Amplicon sizes are shown on the right. Few lanes of the gels were excluded (dotted line). (**b**) Total *Mybpc3* mRNA levels were determined by RT-qPCR with SYBR Green. (**c–e**) The levels of wild-type and mutant *Mybpc3* transcripts were determined by RT-qPCR with specific TaqMan probes. Schemes below show localization of primers (black triangle) and probes. (**f**) Heatmap showing expression of selected genes (threshold <0.7-fold or >1.3-fold change vs. WT EHTs) coding for proteins regulated in hypertrophy, Ca^2+^-handling and K^+^/Na^+^-regulation as well as components of the sarcomere. (**g**) Representative Western blot stained with antibodies directed against FLAG-cMyBP-C and total cMyBP-C. (**h**) Quantification of total cMyBP-C level in single EHTs (WT n = 12; KI-NT n = 10; KI-S282 n = 7; KI-D282 n = 7) normalized to α-actinin and related to WT EHTs. (**i**) Representative blots stained with specific antibodies against Ser-273, Ser-282 and Ser-302 with and without isoprenaline treatment (15 min, 100 nM). α-Actinin served as a cardiac specific loading control. Few lanes of the blots were excluded (dotted lines). Data are expressed as mean ± SEM. **P < 0.01 vs. WT, one-way ANOVA plus Dunnett’s multiple comparisons test (KI-NT, S282 and D282 vs WT EHTs).

To evaluate the molecular impact of phosphomimetic (D282) and wild-type (S282) *Mybpc3* AAV-mediated gene transfer, engineered heart tissues (EHTs) were derived from cardiac cells isolated from neonatal *Mybpc3*-targeted KI mice and transduced with a multiplicity of infection (MOI) of 1,000 vg/cell. After 14 to 16 days of culture, WT, non-transduced KI (KI-NT) and KI EHTs transduced with either S282 (KI-S282) or D282 (KI-D282) AAVs were collected for characterization of the molecular phenotype on mRNA and protein levels (Fig. 1). Qualitative RT-PCR of pooled RNAs showed only one band (=WT) in the WT sample and the expected three bands, corresponding to the different *Mybpc3* mutant transcripts, in the KI-NT sample^15^. In contrast, only the upper band corresponding to WT was still visible in KI EHTs transduced with S282 or D282. A specific band matching FLAG-*Mybpc3* transcript was amplified only in transduced KI EHTs (Fig. 1a). Quantification of total *Mybpc3* mRNA level by RT-qPCR revealed a lower level in KI-NT than in WT (only 25% of WT level), whereas the total *Mybpc3* mRNA level was 6- and 3.5-fold higher in KI-S282 and KI-D282 than in WT, respectively (Fig. 1b). To evaluate the amount of *Mybpc3* WT and mutant mRNAs, a RT-qPCR with specific Taqman probes for WT, missense or frameshift *Mybpc3* mRNAs was performed as previously described^15^. The WT *Mybpc3* transcript was identified in WT as well as in S282- or D282-transduced KI samples but at higher level in the latter two. While no WT *Mybpc3* mRNA was detected in KI-NT samples, the missense *Mybpc3* mRNA was amplified only in KI-NT, but not in *Mybpc3*-transduced KI samples (Fig. 1c,d). Frameshift *Mybpc3* mRNAs were barely detectable in KI-S282 and KI-D282 (Fig. 1e). Expression levels of genes coding for proteins modulating hypertrophy, Ca^2+^/K^+^/Na^+^-handling and constituents of the sarcomere were predominantly higher in KI-NT EHTs, whereas S282 and D282 gene transfer partially restored them to WT levels (Fig. 1f, Suppl. Table S4). In most cases, normalization of gene expression levels was more pronounced after gene transfer with S282 than D282, as in the case of *RyR2* encoding cardiac ryanodine receptor 2.

By Western blotting exogenous FLAG-tagged cMyBP-C protein was detected only in transduced KI EHTs (Fig. 1g). Total cMyBP-C level was significantly lower in KI-NT than in WT EHTs (20% of WT level), corresponding mainly to full-length missense cMyBP-C, whereas no significant difference to WT level was seen in both KI-S282 and KI-D282 EHTs, although high variability were observed between samples (Fig. 1g,h).

To assess the effect of permanent non-reversible phosphorylation-like state of Ser-282 on the phosphorylation level of the other serine residues in the M-motif of cMyBP-C, EHTs were stimulated with 100 nM isoprenaline for 15 min prior to harvesting. Western blots with specific antibodies directed against the three phosphorylated serine residues (pSer-273; pSer-282; pSer-302) showed stronger signals after treatment at all phosphorylation sites especially in the WT and in KI-S282 EHTs. Overall cMyBP-C phosphorylation levels, both at baseline and after treatment, were very low in KI-NT. In KI-D282 EHTs no phosphorylation at Ser-282 neither at baseline nor after isoprenaline stimulation was detected confirming the amino acid substitution and disruption of the pSer-282 epitope. Phosphorylation at Ser-273 was barely detected in KI-NT and transduced samples at baseline and only a faint band was visible after isoprenaline-mediated PKA activation. Ser-302 phosphorylation was similarly increased by isoprenaline stimulation in all groups (Fig. 1i).

### Phosphomimetic cMyBP-C is properly incorporated into the sarcomere

**Figure 2:**
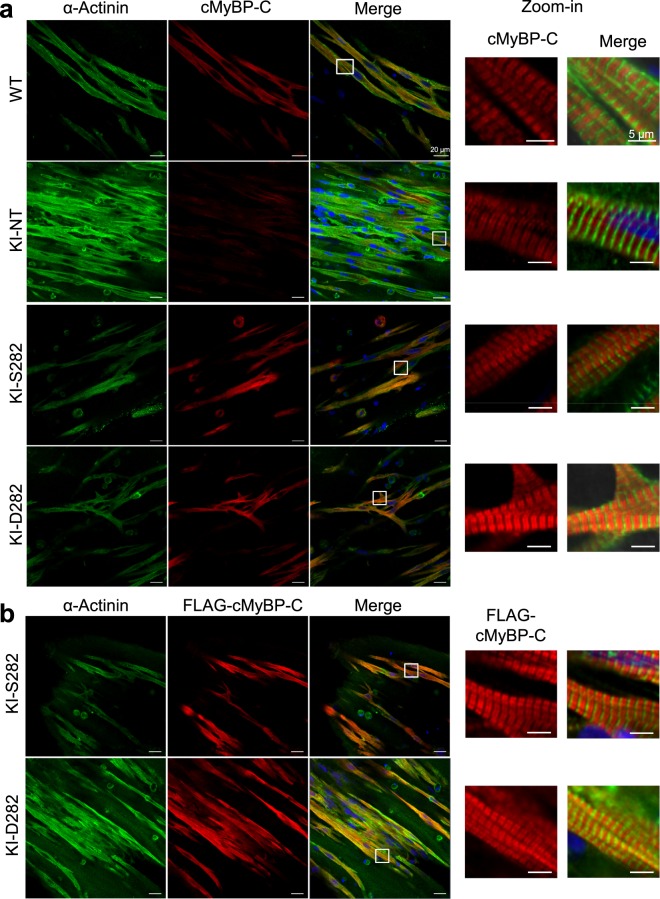
Immunofluorescence images of EHTs of different genotypes. KI EHTs were transduced during casting with AAV6-S282 or -D282 at MOI of 1,000 vg/cell. After 16 days WT, KI-NT, KI-S282 and KI-D282 EHTs were fixed and co-stained with antibodies directed against α-actinin (green) and (**a**) total cMyBP-C (red) or (**b**) exogenous FLAG-tagged cMyBP-C (red). Nuclei were stained with DRAQ5 (blue). Scale bars = 20 µm. Higher magnifications (zoom-in) images for cMyBP-C/FLAG-cMyBP-C and merge are shown on the right side. Scale bars = 5 µm.

The localization of exogenous cMyBP-C proteins was explored by immunofluorescence analyses. The signal intensity of endogenous cMyBP-C was markedly lower in KI-NT than in all other groups, reflecting the lower amount of protein as determined by Western blot (Fig. 2a). Signal intensity did not differ between WT and AAV-transduced KI EHTs. Moreover, almost all α-actinin positive cells were also FLAG-positive, suggesting a nearly complete transduction of EHTs at this MOI. In transduced KI EHTs, the exogenous cMyBP-C protein was well incorporated in the sarcomeres showing doublets at the A-band in regular alternation with α-actinin at the Z-discs (Fig. 2b). Simultaneous staining of FLAG-cMyBP-C and total cMyBP-C showed co-localization (Suppl. Fig. 1).

### Spontaneous contractions are faster in KI EHTs and are normalized with S282 and D282 cMyBP-C gene transfer

**Figure 3:**
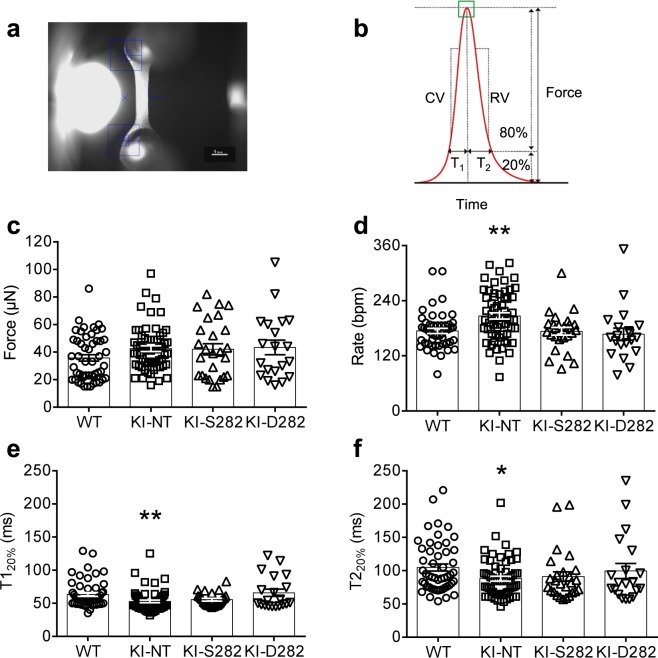
Analysis of contractile parameters in EHTs under spontaneous contraction with video-optical recording. (**a**) A representative image of an EHT as recorded by the video camera within the analysis software and evaluated by automated figure recognition (blue squares). Measurements were performed in EHT culture medium containing 1.8 mM external [Ca^2+^]. (**b**) Schematic contraction peak displaying the evaluated parameters of force, time to contraction (T1_20%_) and time to relaxation (T2_20%_), contraction velocity (CV) and relaxation velocity (RV). (**c**) Maximal forces under spontaneous beating activity of EHTs of different genotypes. Maximal force values were recorded on the day of highest force development. (**d**) Rate of spontaneous beating of EHTs on the day of highest force development (**e**) Contraction time (T1_20%_) and (**f**) relaxation time (T2_20%_) were measured from baseline to 20% of peak height and from peak height to 20% of baseline, respectively, in all EHT groups. Data are expressed as mean ± SEM. Numbers of EHTs/batches in all panels: WT 51/5; KI-NT 61/7; KI-S282 26/5; KI-D282 20/5. *P < 0.05 and **P < 0.01 vs. WT, one-way ANOVA plus Dunnett’s multiple comparisons test (KI-NT, S282 and D282 vs WT EHTs).

We then evaluated the impact of D282 and S282 *Mybpc3* AAV-mediated gene transfer on contractile parameters of EHTs. EHTs generated from *Mybpc3* WT and KI mouse cardiac cells showed a similar development after viral transduction. Beating areas within the murine EHTs were visible already 3 days after casting and coherent contractions of the whole EHTs with deflection of the posts resulting in generation of measurable forces at day 5. Generally, maximal force development was reached between days 12–16 and continuously declined thereafter in every group independently of the genotype or viral transduction (data not shown). Therefore, video-optical recording of contractile parameters was performed between days 13 and 15 (Fig. 3a,b, Table 1). As quality control, only EHTs beating with a frequency of ≥50 bpm and producing forces ≥15 µN were taken into account for the analysis. Spontaneous beating activity in each group was measured in EHT culture medium containing 1.8 mM external [Ca^2+^]. In this condition, KI-NT developed a tendency towards higher force than WT EHTs, although non-significant with one-way ANOVA. However, with the Student’s t-test force was higher in KI-NT than in WT EHTs (*P* < 0.05). Transduction of KI EHTs with either S282 or D282 AAV did not affect maximal force development (Fig. 3c; Table 1). The beating frequency on the day of maximal force development was recorded in all groups and showed a Gaussian distribution. KI-NT beat at significantly higher frequencies than WT EHTs (Table 1), whereas viral transduction with either S282 or D282 construct in KI EHTs normalized beating rate to WT EHTs (Fig. 3d; Table 1). Contraction time (T1_20%_) and relaxation time (T2_20%_) were significantly shorter in KI-NT than in WT (−17% and −16%, respectively) and did not differ between transduced KI and WT EHTs (Fig. 3e,f; Table 1). Even though contraction velocity (CV) and relaxation velocity (RV) showed higher values for KI-NT, they were not significantly different between the groups (Suppl. Fig. 2a,b).

**Table 1:** Contractile parameters in EHTs under spontaneous contraction.

	WT	KI-NT	KI-S282	KI-D282
Number of values	51	61	26	20
Force (µN)	35.73 ± 2.32	42.52 ± 2.06	42.04 ± 4.10	43.4 ± 5.28
Rate (bpm)	174 ± 6	207 ± 7**	173 ± 8	167 ± 13
Contraction velocity (µN/ms)	0.89 ± 0.06	1.06 ± 0.06	0.99 ± 0.10	1.01 ± 0.12
T1_20%_ (ms)	63.41 ± 2.86	52.84 ± 1.99**	55.46 ± 1.94	65.80 ± 5.57
Relaxation velocity (µN/ms)	0.70 ± 0.05	0.83 ± 0.05	0.78 ± 0.07	0.82 ± 0.1
T2_20%_ (ms)	104.70 ± 5.38	87.98 ± 3.63*	91.00 ± 7.23	99.50 ± 11.19

Data are expressed as mean ± SEM. Groups were compared with the one-way ANOVA, followed by Dunnet’s multiple comparison test vs WT. *P < 0.05 and ** *P* < 0.01 vs WT EHTs.

### Electrical pacing reveals hypercontractility in KI EHTs, which is similarly prevented by S282 and D282 cMyBP-C gene transfer

**Figure 4:**
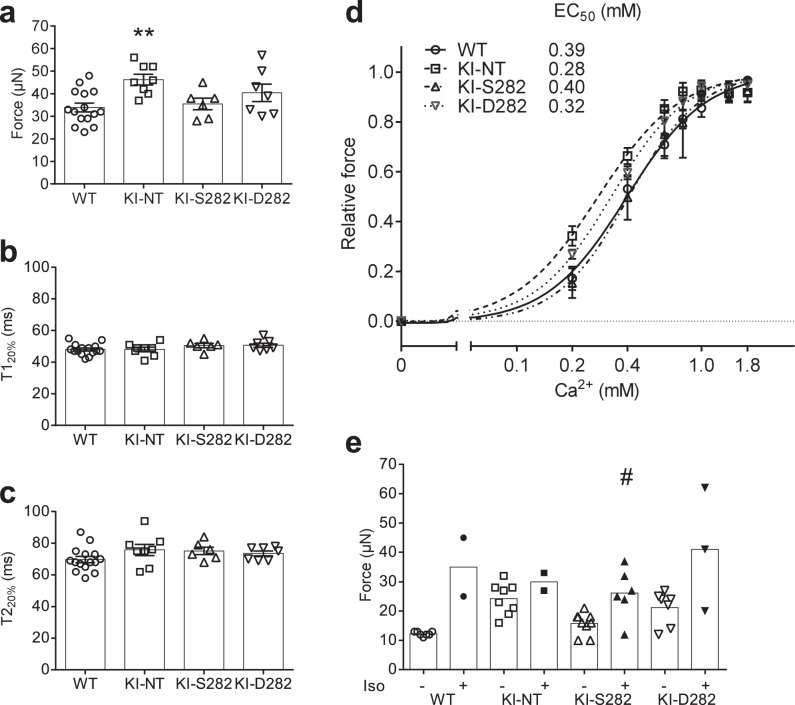
Contractility analysis of EHTs under electrical stimulation. EHTs were exposed to Tyrode’s solution containing different external [Ca^2+^] (0.1–1.8 mM) and paced at a murine physiological frequency (6 Hz) at culture days 14–16. (**a**) Maximal force of contraction of the different groups at 1.8 mM external [Ca^2+^] and (**b**) corresponding contraction time (T1_20%_) and (**c**) relaxation time (T2_20%_). (**d**) Relative force-[Ca^2+^] curves with calculated EC_50_ values. (**e**) Effect of 100 nM isoprenaline (Iso) on force at submaximal [Ca^2+^] (0.4 mM). Data are expressed as mean ± SEM. WT n = 15; KI-NT n = 8; KI-S282 n = 6; KI-D282 n = 7. **P < 0.01 vs. WT one-way ANOVA plus Dunnett’s multiple comparisons test (KI-NT, S282 and D282 vs WT EHTs). ^#^*P* < 0.05 vs. corresponding baseline, paired Student’s t-test (panel e). Concentration response curves were fitted to the data points and curve comparison was done by using extra sum-of-squares F-test, log-EC_50_ was different for each data set. *P* = 0.0008 (panel d).

Since in this study the known hypercontractile phenotype (i.e. higher force, accelerated velocities of contraction and relaxation) of KI-NT EHTs was blunted under spontaneous beating due to the increased beating rate (negative force-frequency relationship), EHTs of all genotypes were subjected to electrical pacing at a murine physiological frequency of 6 Hz^17,28^. At 1.8 mM external [Ca^2+^], KI-NT showed 1.4-fold higher maximal force than WT EHTs (Fig. 4a; Table 2). Maximal force of contraction was lower in KI-S282 than in KI-NT EHTs (*P* < 0.05 with Student’s t-test) and did not significantly differ from WT EHTs. On the other hand, although the mean maximal force developed by KI-D282 EHTs was halfway between KI-NT and WT EHTs, there was a high variability among samples, excluding significant difference from the other groups (Fig. 4a; Table 2). No major differences in contraction (T1_20%_) and relaxation (T2_20%_) times were measured in the different groups (Fig. 4b,c; Table 2). Accordingly, contraction and relaxation velocities matched the measured forces (Suppl. Fig. 2c,d; Table 2). In addition to contractile parameters, the sensitivity of the different groups toward external [Ca^2+^] was investigated. KI-NT EHTs exhibited a left shift of the force-calcium curve, indicating higher [Ca^2+^] sensitivity than the WT (EC_50_ of 0.28 vs 0.39 mM, respectively). After transduction, both KI-S282 and KI-D282 presented a right shift of the force-calcium curve, which was less pronounced in KI-D282 EHTs (EC_50_ of 0.40 and 0.32 mM, respectively). The Hill coefficient did not differ between the groups (WT 2.31 ± 0.31, KI-NT 2.02 ± 0.23, KI-S282 2.51 ± 0.35, KI-D282 2.16 ± 0.20; Fig. 4d). Comparison among the groups at each single [Ca^2+^] with one-way ANOVA plus Dunnett’s test revealed a significant difference only between KI-NT and WT EHTs at low calcium (0.2 mM). Furthermore, EHTs of all genotypes were treated with 100 nM isoprenaline at submaximal external [Ca^2+^] in Tyrode’s solution and paced at 6 Hz. After 15 min of incubation, contractile measurements showed a positive inotropic effect in WT EHTs (2.8-fold increased force vs. baseline), whereas the response was blunted in KI-NT EHTs (1.2-fold increased force vs. baseline; Fig. 4e). Gene transfer with S282 and D282 partially restored the isoprenaline-response of KI-NT to WT level (1.7-fold increased force vs. baseline). In addition, WT, KI-S282 and KI-D282 EHTs showed a shorter relaxation time (T2_20%_) fitting well with the positive lusitropic effect of isoprenaline (Suppl. Fig. 3). However, during the treatment many EHTs, independently of the genotype, became arrhythmic and were excluded from the analysis.

**Table 2:** Contractile parameters in paced EHTs.

	WT	KI-NT	KI-S282	KI-D282
Number of values	8	15	6	7
Force (µN)	33.87 ± 1.95	46.25 ± 2.34**	35.50 ± 2.59	40.43 ± 3.87
Contraction velocity (µN/ms)	1.11 ± 0.06	0.85 ± 0.05**	0.81 ± 0.06	0.95 ± 0.0.08
T1_20%_ (ms)	48.00 ± 0.92	48.00 ± 1.43	50.50 ± 1.33	50.71 ± 1.32
Relaxation velocity (µN/ms)	0.81 ± 0.08	0.67 ± 0.04	0.65 ± 0.09	0.70 ± 0.05
T2_20%_ (ms)	69.73 ± 1.96	75.75 ± 3.54	75.17 ± 2.36	73.57 ± 1.54

Data are expressed as mean ± SEM. Groups were compared with the one-way ANOVA, followed by Dunnet’s multiple comparison test vs WT. **P < 0.01 vs WT EHTs.

## Discussion

It has been shown previously that substitution of the cMyBP-C serine phosphorylation sites with aspartic acid residues (mimicking activated protein) protects against ischemia-reperfusion injury^21^, whereas substitution with alanine residues (mimicking deactivated protein) results in cardiac hypertrophy with myofibrillar disarray and fibrosis in mice^33^. Overall cMyBP-C phosphorylation levels are reduced in HCM patients and animal models^34–36^, thus increasing cMyBP-C phosphorylation could be a therapeutic option. The goal of this study was to evaluate whether gene therapy with a phosphomimetic cMyBP-C protein (D282) would be more beneficial than wild-type cMyBP-C protein (S282) to prevent the molecular and functional disease phenotype in a well-established EHT model derived from *Mybpc3*-targeted KI mice. The main findings of this work are as follows: i) AAV6-mediated transduction with D282 or S282 similarly suppressed accumulation of mutant *Mybpc3* transcripts and restored cMyBP-C protein levels. ii) Both exogenous cMyBP-C proteins were correctly incorporated into the sarcomere in KI EHTs. iii) Spontaneous contractions were faster in KI EHTs and were normalized by S282 and D282 cMyBP-C gene transfer. iv) Electrical stimulation at murine physiological frequency revealed hypercontractility in KI EHTs, which was prevented at least by S282 cMyBP-C gene transfer. v) The effect of S282 on normalization of the force-Ca^2+^-relationship and expression of genes encoding proteins involved in hypertrophy, Ca^2+^-handling, K^+^/Na^+^-regulation and sarcomere components was stronger than with D282.

Phosphorylation is a reversible post-translational modification. To investigate its role in protein function a new protein can be engineered carrying an aspartic acid (D) instead of serine (S; here D282 vs S282 cMyBP-C). This substitution introduces a negative charge similarly to the phosphoryl group, however D is smaller and have a different geometry therefore the engineered protein should not be addressed as constitutively phosphorylated. The resulting protein might be considered as a gain of function being a constitutively active protein. AAV6-mediated gene transfer of KI-NT EHTs with either D282- or S282-*Mybpc3* resulted in higher amounts of *Mybpc3* mRNAs than in WT EHT, but same protein levels. In addition, both exogenous proteins were correctly incorporated in the sarcomere. That sarcomeric proteins are maintained in a strict stoichiometric balance is well known^37^. It has been shown that mRNA transcripts, translation and protein degradation of sarcomeric components occur directly on site at the sarcomere and, by working in unison, these mechanisms ensure correct spatial localization and overcome the large variability in transcription of sarcomeric proteins^38^. Therefore, the stoichiometric maintenance is regulated at all steps from gene transcription to protein degradation. Our molecular data are in agreement with previous findings obtained in KI EHT^25^ and in EHTs derived from *Mybpc3*-targeted knock-out mice (KO EHTs^17^). We also confirmed in the present study that gene transfer with a full length *Mybcp3* sequence prevents the accumulation of mutant transcripts in KI EHT and showed that it did not differ between the two types of construct. Therefore, both treatments have the same effect on haploinsufficiency and poison polypeptides, the two pathomechanisms involved in *Mybpc3*-related cardiomyopathy.

Cardiac MyBP-C, located transversally in doublets in the C-zone of the A-bands, acts as a “brake”, a structural barrier for the cross-bridge cycling preventing the binding of myosin heads to the thin filaments^39^. Thus, cMyBP-C stabilizes a population of myosin heads in the inactive super-relaxed state (SRX), keeping them closely bound to the thick filament and unavailable to generate force^40^. Another molecular model is that the N-terminal domains of cMyBP-C modulates the thin filament activity by displacing tropomyosin and therefore enhancing myosin binding to actin^41,42^. Beside reducing the affinity of the N-terminal domains for the thin filaments^43^, phosphorylation of cMyBP-C at Ser-282 plays a primary role in SRX regulation, releasing myosin heads from the inhibitory state^44^. Low level or absence of cMyBP-C is expected to untether the myosin heads and relieves them from the SRX, thereby enhancing contraction. In KI cardiomyocytes, the level of cMyBP-C (corresponding mainly to missense protein) represents only 10% of its level in WT. Compared to the normal situation, more myosin heads can bind to actin resulting in more force generation. It has been reported before that EHTs derived from *Mybpc3*-targeted KI and KO mice exhibit higher maximal force development, accelerated rates of both contraction and relaxation and higher sensitivity to external [Ca^2+^] compared to WT EHTs (=hypercontractile phenotype), both under spontaneous contraction and electrical stimulation^17,25,28,45^. In the present work, one-way ANOVA comparison did not reveal higher force, but shorter contraction and relaxation times and higher bpm in KI-NT EHTs under spontaneous contraction (Fig. 3; Table 1). However, significant higher force in KI-NT was revealed by applying a Student’s t-test between KI-NT and WT EHTs. Pacing unmasked the hypercontractile phenotype (higher force of contraction) of KI EHTs without change in T1_20%_ and T2_20%_ (Table 2). Therefore, this model revealed either hypercontractility under pacing or faster kinetics of contraction and relaxation under spontaneous contraction. The faster relaxation was not expected since we previously reported prolonged relaxation in intact adult KI cardiomyocytes and diastolic dysfunction *in vivo*^35^. On the other hand, our data are in line with accelerated kinetics of contraction and/or higher force development in *Mybpc3* KO EHTs^28,45,46^ and in *Mybpc3* KI EHTs^17^. The discrepancy between the data obtained in EHT and in cardiac myocytes or *in vivo* in KI mice could be related to the different experimental conditions and/or age of the mice. Indeed, in EHT, cardiac cells are derived from neonatal mice and the muscle strips develop force against silicon posts in an auxotonic manner. On the contrary, intact cardiomyocytes derived from adult heart contract in an isotonic manner. Moreover, EHTs as an *in vitro* model do not present remodeling of the myocytes or of the entire tissue such as *in vivo* in the whole heart. The prevention of the EHT disease phenotype after *Mybpc3* D282 and S282 gene transfer was expected in both conditions since the amount of cMyBP-C protein was completely restored, and we recently showed that ≥73% of wild-type cMyBP-C in a *Mybpc3* null background is enough to overcome the disease phenotype^17^. Increased myofilament [Ca^2+^] sensitivity is a well-accepted hallmark of HCM as seen in human samples^11,36,47,48^ and in HCM animal models^15,17,35,49–53^. Accordingly, KI-NT showed higher sensitivity to external [Ca^2+^] than WT EHTs. AAV-mediated *Mybpc3* gene transfer reduced the sensitivity to external [Ca^2+^] towards WT values. However, the effect of D282 was not as pronounced as with S282. A nonspecific effect of transduction *per se* was ruled out with an empty virus (only promoter, no transgene; data not shown).

Isoprenaline cannot give rise to phosphorylation of the D282 but the S282-cMyBP-C. Ser-282 is a highly conserved amino acid in cMyBP-C and located in the vicinity of three more serine residues (Ser-273, Ser-302 and Ser-307) in the M-motif of the protein. These residues are targets of several protein kinases for phosphorylation following β-adrenergic stimulation^6^. Several groups have tried to decipher the role of each single cMyBP-C phosphorylation site and their interplay using transgenic mice generated *ad hoc*^22,23,54,55^. While it is well accepted that phosphorylation of Ser-282 plays a leading role, its effect on phosphorylation of the other residues in the motif is not completely clear^22,23^. In our study, while isoprenaline stimulation induced a similar, but strong phosphorylation at Ser-302 in both gene therapy approaches, the effect on Ser-273 was clearly smaller, suggesting that beside PKA other kinases might phosphorylate this residue. Isoprenaline showed a positive inotropic effect in all groups except KI-NT EHTs. The blunted response to the drug in the latter is in line with previous findings^28^, and may be due to the fact that most of the cMyBP-C-mediated brake is absent in KI and basal force is already higher. Phosphorylation of cMyBP-C induces bending of the M-motif and structural changes of the N-terminus of the protein, which reveals a stretch of amino acid residues that could be part of a yet-unknown protein-protein interaction site^43^. The PKA-mediated phosphorylation of cMyBP-C could result in increased myosin binding to the thin filament or stronger activation of thin filaments, in both cases ultimately leading to a stronger positive inotropic effect^56^.

Interestingly, the main difference between the two treatments concerned gene expression. Up-regulation of several genes involved in hypertrophic signaling pathway (e.g. *Myh7*), Ca^2+^-handling (e.g. *Cacna1c* and *Ryr2*), K^+^/Na^+^-regulation (e.g. *Kcnip2*) and sarcomere function (e.g. *Des*) present in KI-NT was prevented by both treatments, but to a lower extent with D282. This only partial correction of gene expression after D282 gene transfer could contribute to the smaller impact on the reduction of the hypercontractile KI-NT EHT phenotype of D282 compared to S282 EHTs, since more L-type calcium channels and ryanodine receptors would result in more intracellular [Ca^2+^] and therefore contraction.

## Limitations of the Study

The number of paced EHTs included in the analyses was low, particularly after isoprenaline treatment, making impossible any powerful statistical conclusion. Nonetheless, our data are consistent with previous findings showing a lower positive inotropic response in KI-EHTs than in WT-EHTs after isoprenaline treatment^28^. A second limitation is the phenotype of KI EHTs *per se*. This *in vitro* three-dimensional model derived from cardiac cells of KI mice showed higher force at baseline compared to WT. In contrast, *in vivo* echocardiographic analysis of KI mice showed impaired cardiac function, as revealed by lower fractional area shortening and E’/A’ ratio^15,35^, resembling more the cardiac phenotype seen in HCM patients. In addition, it should be noted that whereas the majority of *MYBPC3*-related HCM patients carries the mutation at the heterozygous state, the KI-EHT used in the present study are derived from mice carrying the *Mybpc3* mutation at the homozygous state. As a matter of fact the heterozygous *Mybpc3* knock-in mice, which would reflect the human genetic state, do not develop left ventricular hypertrophy^15,35^.

## Conclusion

KI EHTs exhibited hypercontractility, which was similarly prevented by AAV-mediated transduction of wild-type S282 or phosphomimetic D282 cMyBP-C. However, the impact of D282 on normalization of the force-Ca^2+^ relationship and gene expression profile of proteins modulating hypertrophy, Ca^2+^-, K^+^/Na^+^-handling and constituents of the sarcomere was not as pronounced as for S282, even though protein steady-state levels and localization did not differ. Future work has to explore whether the advantage of S282 compared to D282 in preventing the HCM phenotype extends to *in vivo* situations.

## Supplementary information

Supplementary Information
