# Supplementary material for: Phosphomimetic cardiac myosin-binding protein C partially rescues a cardiomyopathy phenotype in murine engineered heart tissue
Source: Sci Rep. 2019 Dec 3;9:18152. doi: 10.1038/s41598-019-54665-2 (PMC6890639; doi:10.1038/s41598-019-54665-2)
Supplement: Supplementary file 1 — Supplementary Information [file 41598_2019_54665_MOESM1_ESM.pdf]

Supplementary Information

Phosphomimetic cardiac myosin-binding protein C partially rescues a cardiomyopathy phenotype in murine engineered heart tissue

Alexander Dutsch[1,2,3,4], Paul J.M. Wijnker[1,2,5], Saskia Schlossarek[1,2], Felix W. Friedrich[1,2], Elisabeth Krämer[1,2], Ingke Braren[1,2,6], Marc N. Hirt[1,2], David Brenière-Letuffe[1,2], Alexandra Rhoden[1,2], Ingra Mannhardt[1,2], Thomas Eschenhagen[1,2], Lucie Carrier[1,2*], Giulia Mearini[1,2*]

[1]Institute of Experimental Pharmacology and Toxicology, Cardiovascular Research Center, University Medical Center Hamburg-Eppendorf, Hamburg, Germany;

[2]DZHK (German Centre for Cardiovascular Research), partner site Hamburg/Kiel/Lübeck, Hamburg, Germany;

[3]Department of internal Medicine III, University Hospital Schleswig-Holstein, University of Kiel, Kiel, Germany;

[4]DZHK (German Centre for Cardiovascular Research), partner site Hamburg/Kiel/Lübeck, Kiel, Germany;

[5]Department of Physiology, Amsterdam Cardiovascular Sciences, Amsterdam UMC, location VUmc, Amsterdam, The Netherlands;

[6]Vector Core Unit, Institute of Experimental Pharmacology and Toxicology, University Medical Center Hamburg-Eppendorf, Hamburg, Germany

Suppl. Table S1: Primer sequences for site-directed mutagenesis

Primer	Sequence (5' to 3')
Mybpc3 S282D F	GAGCAGGTCGGAGAACCGATGACAGCCATGAAGATG
Mybpc3 S282D R	CATCTTCATGGCTGTCATCGGTTCTCCGACCTGCTC

Suppl. Table S2: Primer sequences for RT-PCR and RT-qPCR with SYBR-Green

Primer	Sequence (5' to 3')
FLAG F	GGATTACAAGGATGACGACGA
Gapdh F	ATTCAACGGCACAGTCAAG
Gapdh R	TGGCTCCACCCTTCAAGT
Mybpc3 ex.1 F	CACCCCTGGTGTGACTGTTCTCAA
Mybpc3 ex.2 R	GTCATCAGGGCTCGCATC
Mybpc3 ex.2 R	CTGACCGCTCCGTCTCAG
Mybpc3 ex.4 F	TCTTTCTGATGCGACCACAG
Mybpc3 ex.9 R	TCCAGAGTCCCAGCATCTTC

Suppl. Table S3: Primers and probes sequences for RT-qPCR

Gene acronym	Full name	Primers/Probe	Sequence (5' to 3')
Gnas	*Guanine nucleotide-binding protein, alpha stimulating*	F	CAAGGCTCTGTGGGAGGAT
		R	CGAAGCAGGTCCTGGTCACT
		Probe	FAM-GCTGATTGACTGTGCCCAGTACTTCCT-TAMRA
Mybpc3	*Myosin-binding protein C, cardiac*	WT/Mut1 F	GTGTCTACCAAGGACAAATTTGACA
		WT/Mut1 R	CCAGGTCTCCAGAACCAATG
		WT Probe	FAM-AACCTCACTGTCCATGAG-MGB
		Mut1 Probe	VIC-CTCACTGTCCATAAGG-MGB
		Mutant 2/3 F	TGGACCTGAGCAGCAAAGTG
		Mutant 2/3 R	GGTCCAGGTCTCCAGAACCA
		Mutant 2/3 Probe	FAM-CCAGCAAGAGGCCA-MGB

Suppl. Table S4: Gene acronym, full name and expression level in WT, KI-NT, KI-S282 and KI-D282 EHTs

	Gene acronym	Gene full name	WT	KI-NT	KI-S282	KI-D282
Hypertrophy	*Actc1*	*Actin, alpha, cardiac muscle 1*	1.00	2.03	1.32	1.49
	Ctgf	*Connective tissue growth factor*	1.00	1.39	1.22	1.21
	Fhl1	*Four-and-a-half-LIM-domains 1*	1.00	1.78	1.16	1.49
	Meox	*Mesenchyme homeobox 1*	1.00	2.16	2.21	2.13
	Myh7	*Myosin, heavy polypeptide 7, cardiac muscle, beta*	1.00	3.58	1.24	2.63
	Nppb	*Natriuretic peptide B*	1.00	1.94	0.65	1.04
	Rcan1	*Regulator of calcineurin 1*	1.00	1.63	1.05	1.30
Ca^{2+} handling	*Cacna1c*	*Calcium channel, voltage-dependent, L type, alpha 1C subunit*	1.00	1.44	1.20	1.29
	Cacna1g	*Calcium channel, voltage-dependent, T type, alpha 1G subunit*	1.00	1.57	1.82	1.36
	Gja1	*Gap junction protein, alpha 1, 43kDa*	1.00	1.47	1.07	1.16
	Pln	*Phospholamban*	1.00	1.44	1.39	1.42
	Ryr2	*Ryanodine receptor 2, cardiac*	1.00	11.33	1.17	6.02
K+/Na+ regulation	*Kcnb1*	*Potassium voltage gated channel, Shab-related subfamily, member 1*	1.00	1.36	1.46	1.40
	Kcnip2	*Kv channel-interacting protein 2*	1.00	3.55	0.78	2.17
	Kcnj2	*Potassium inwardly-rectifying channel, subfamily J, member 2*	1.00	nd	1.17	nd
	Scn5a	*Sodium channel, voltage-gated, type V, alpha*	1.00	1.39	1.10	1.54
Sarcomere	*Actn2*	*Actinin alpha 2*	1.00	1.47	1.30	1.32
	Cryab	*Crystallin, alpha B*	1.00	1.57	1.28	1.41
	Csrp3	*Cysteine and glycine-rich protein 3*	1.00	1.61	1.27	1.55
	Des	*Desmin*	1.00	2.53	1.07	1.42
	Flnc	*FilaminC*	1.00	1.48	0.95	1.43
	Ldb3	*LIM domain binding 3*	1.00	1.69	1.36	1.46
	Myl2	*Myosin, light polypeptide 2, regulatory, cardiac*	1.00	1.71	1.75	1.76
	Myl3	*Myosin, light polypeptide 3, essential, cardiac*	1.00	1.69	1.48	1.54
	Myoz2	*Myozenin 2*	1.00	1.60	1.44	1.36
	Mypn	*Myopalladin*	1.00	2.18	1.45	1.20
	Myzap	*Myocardial zonula adherens protein*	1.00	2.05	1.34	1.24
	Nexn	*Nexilin*	1.00	1.52	1.28	1.49
	Obscn	*Obscurin*	1.00	1.53	1.17	1.77
	Tcap	*Telethonin*	1.00	1.34	1.87	1.41
	Tnnc1	*Troponin C, cardiac*	1.00	1.46	1.31	1.33
	Ttn	*Titin*	1.00	1.62	1.29	1.60

Values in KI-NT, KI-S282 and KI-D282 are given in fold-change over WT. nd: not-determined.

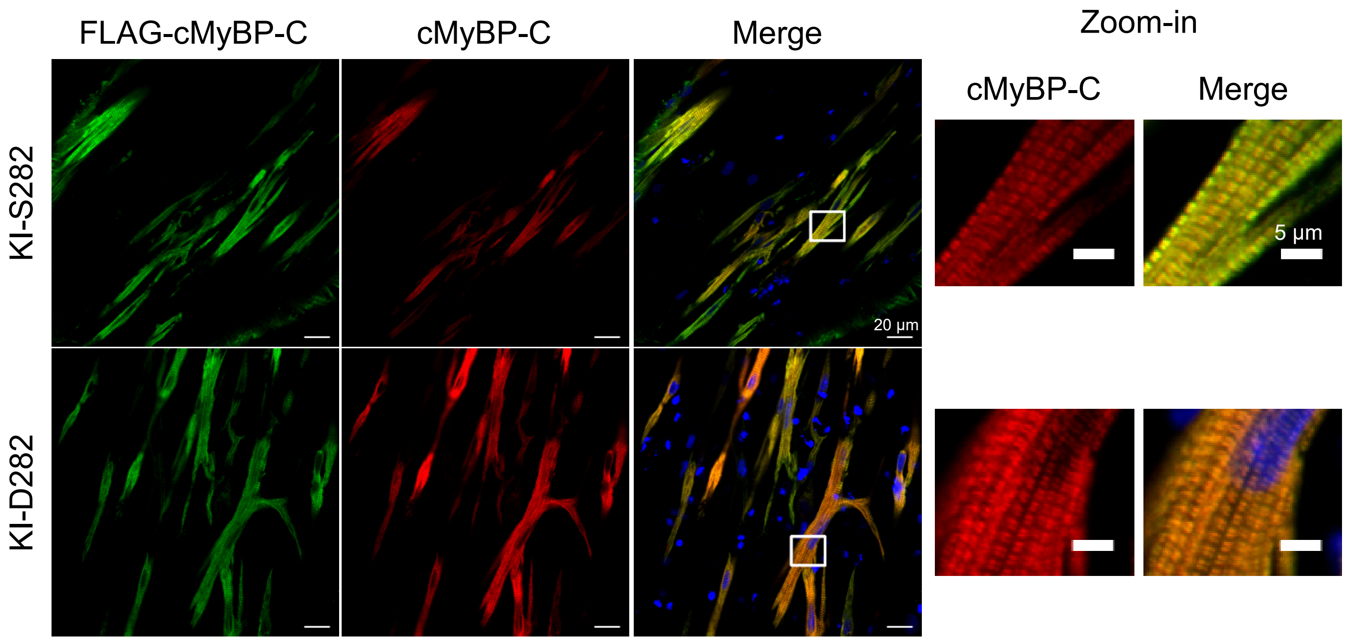

Suppl. Fig. 1 Immunofluorescence images of KI EHTs transduced with AAV6 encoding wild-type cMyBP-C (S282) or phosphomimetic cMyBP-C (D282) KI EHTs were transduced at MOI of 1,000 vg/cell. After fixation EHTs were co-stained with antibodies directed against the FLAG-tag for exogenous cMyBP-C (green) and total cMyBP-C (red). Nuclei were stained with DRAQ5 (blue). Scale bars 20 µm. Higher magnifications (zoom-in) images are shown on the right side. Scale bars 5 µm.

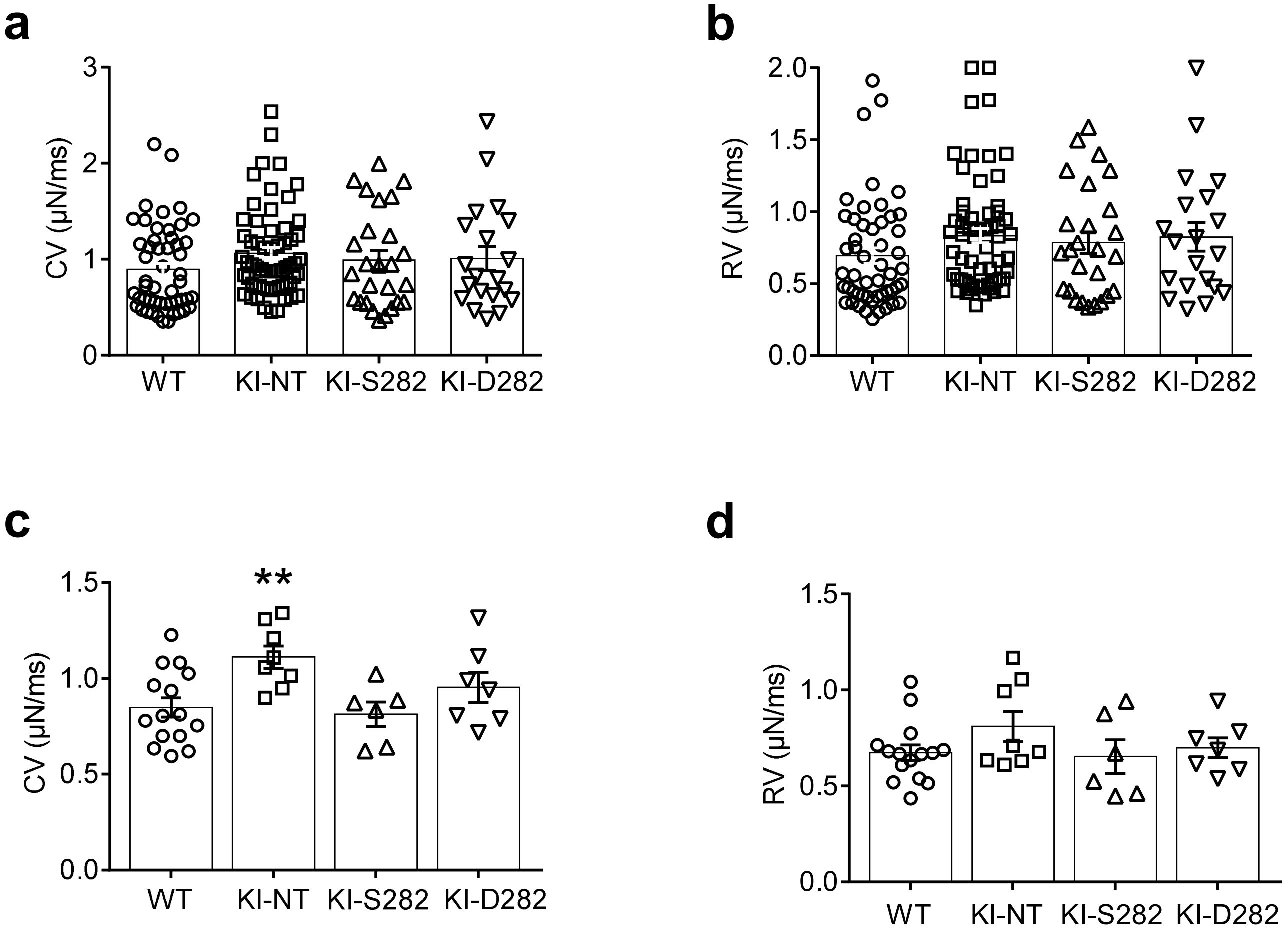

Suppl. Fig. 2 Contraction velocity (CV) and relaxation velocity (RV) measured on the day of highest force development [a]-[b] Contraction and relaxation velocities under spontaneous contraction. Numbers of EHTs/batches: WT 51/5; KI-NT 61/7; KI-S282 26/5; KI-D282 20/5. **[c]-[d]** Contraction and relaxation velocities calculated under electrical stimulation. WT n = 15; KI-NT n = 8; KI-S282 n = 6; KI-D282 n = 7. Data are expressed as mean±SEM. ***P*<0.01 vs. WT, one-way ANOVA plus Dunnett's multiple comparisons test.

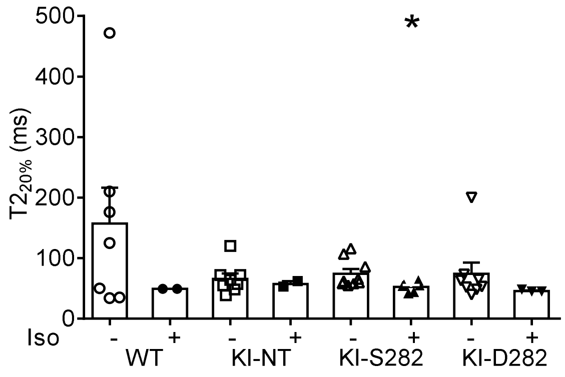

Suppl. Fig. 3 Effect of isoprenaline on relaxation time ($T2_{20\%}$) under electrical stimulation. EHTs of all groups were treated 15 min with 100 nM isoprenaline at submaximal external [Ca^{2+}] in Tyrode's solution and paced at 6 Hz. $T2_{20\%}$ was decreased in WT, S282 (*P <0.05 Student's t-test) and D282 EHTs.

Full agarose gels. Red rectangles show the selected part, red arrow shows displayed bands in the figures

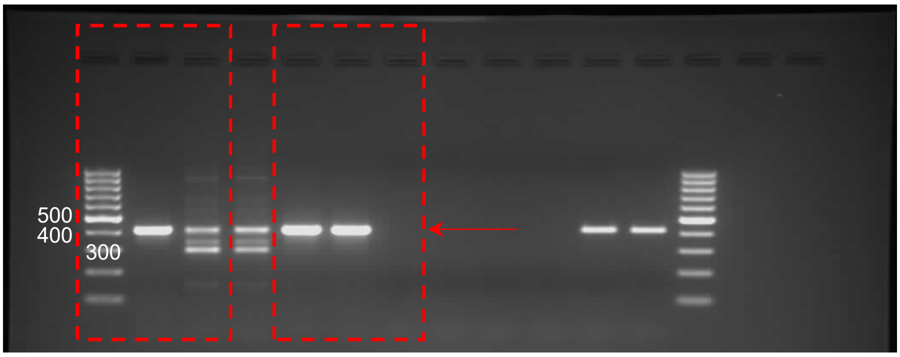

Fig. 1a upper panel

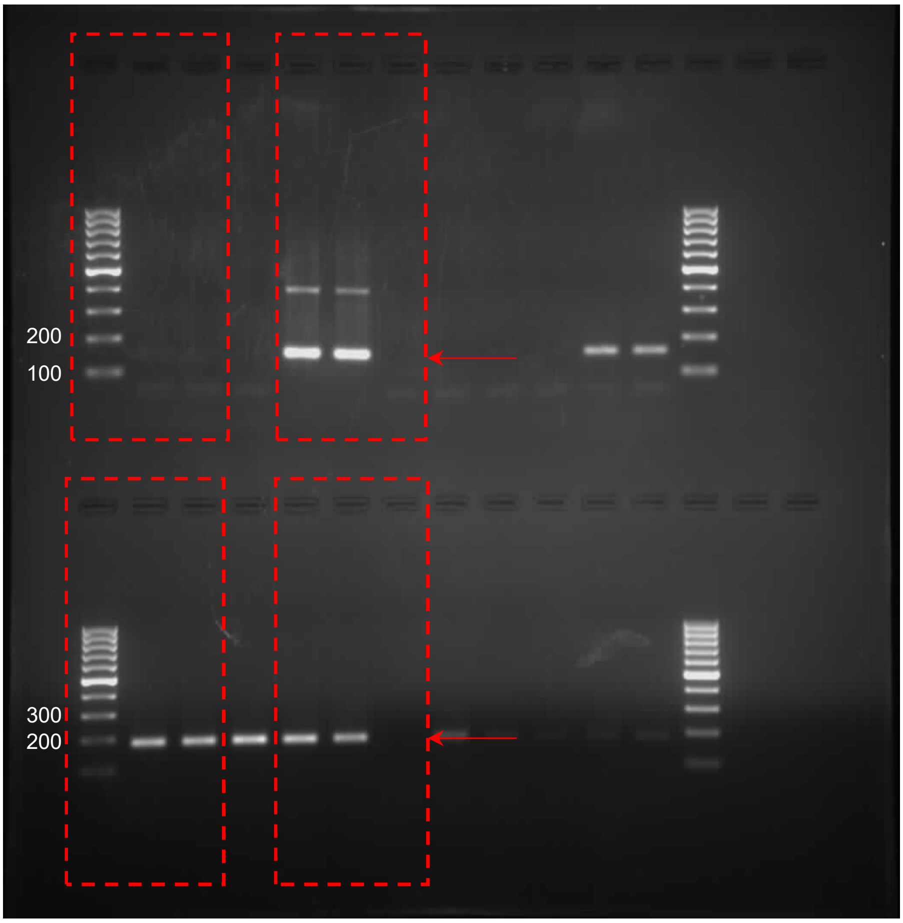

Fig. 1a middle panel

Fig. 1a bottom panel

Full Western blot membranes. Red rectangles show the selected part, red arrow shows displayed bands in the figures

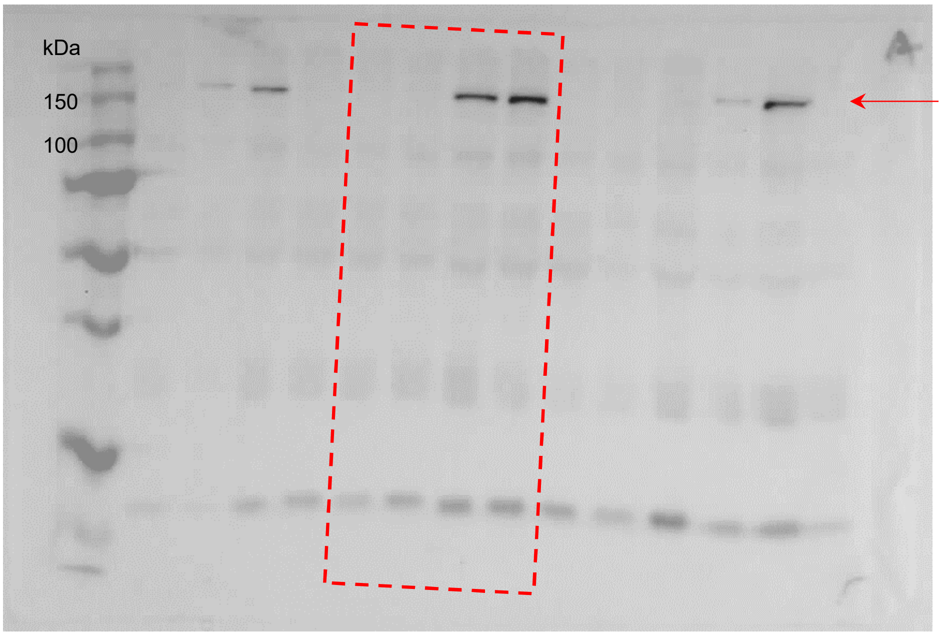

Fig.1g FLAG

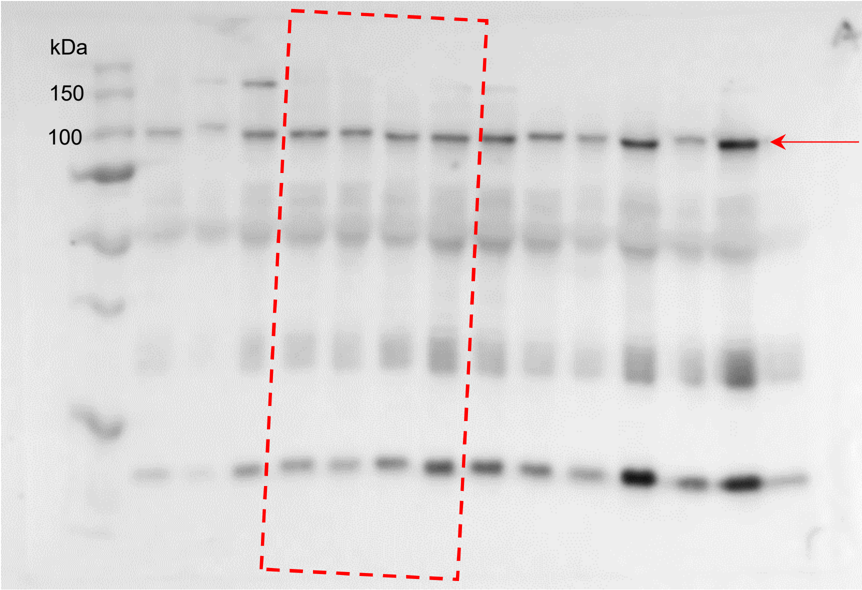

Fig.1g α-Actinin

Full Western blot membranes. Red rectangles show the selected part, red arrow shows displayed bands in the figures

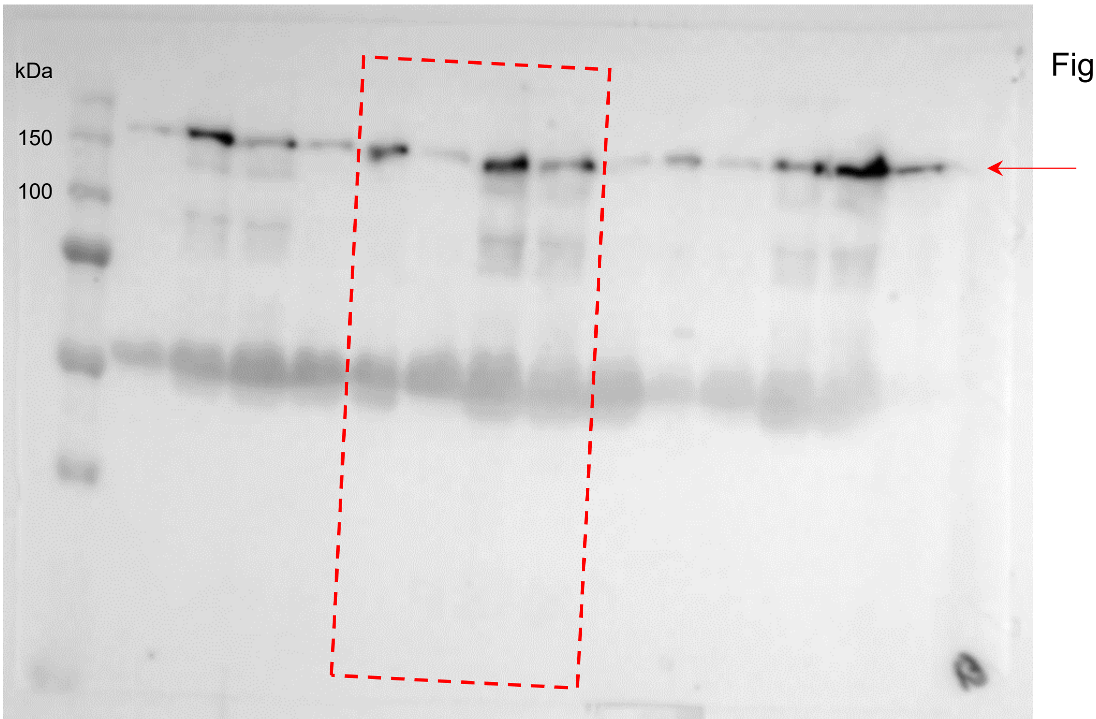

Fig.1g cMyBP-C

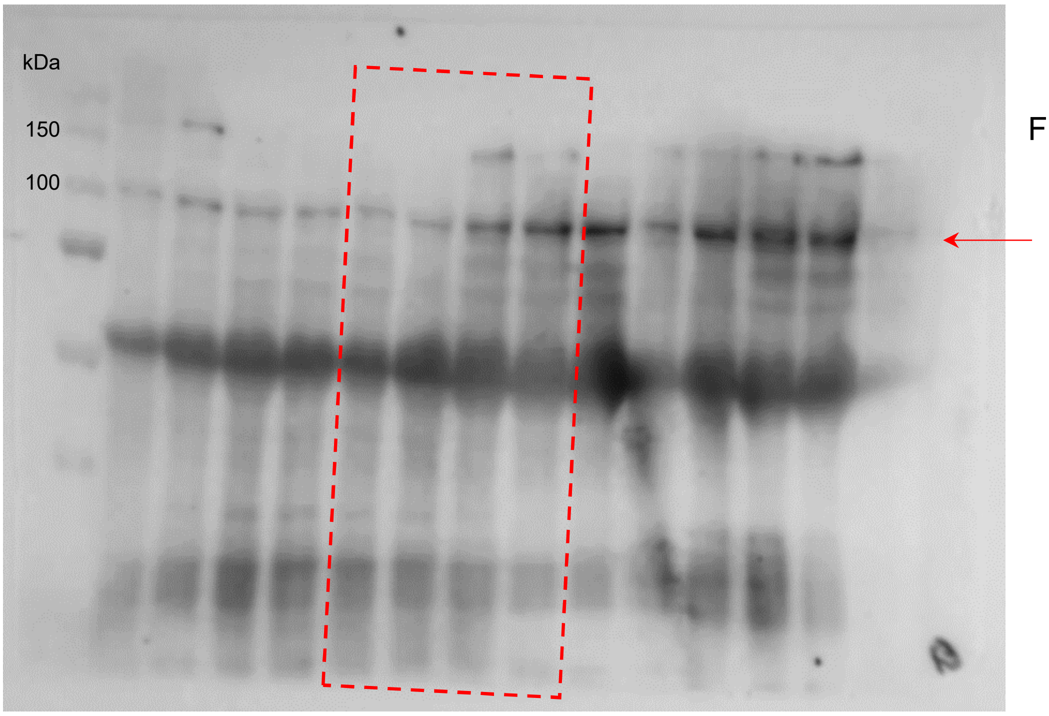

Fig.1g α-Actinin

Full Western blot membranes. Red rectangles show the selected part, red arrow shows displayed bands in the figures

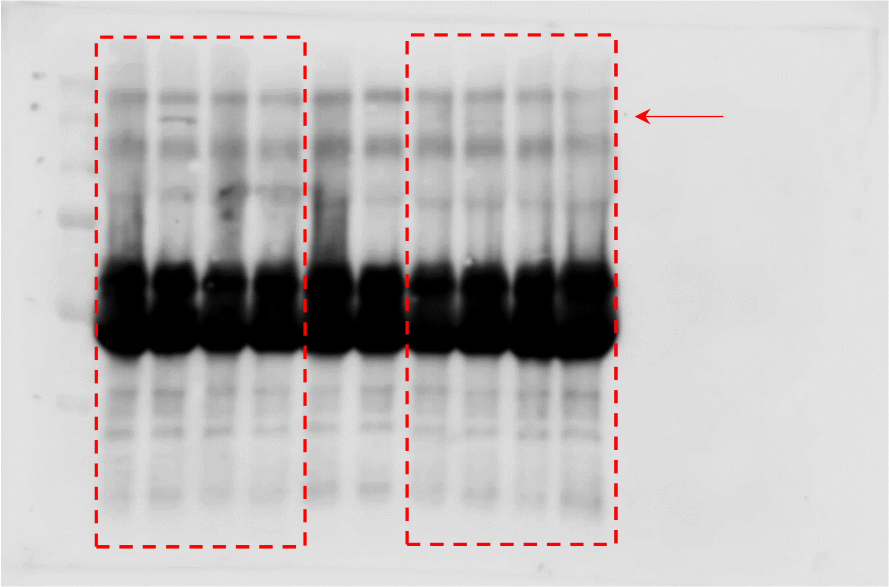

Fig.1i pSer-273 cMyBP-C

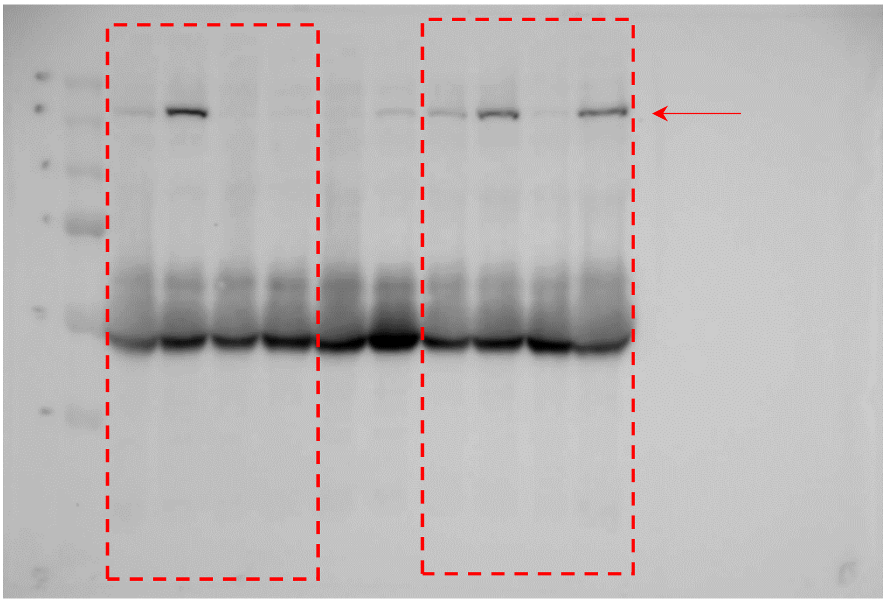

Fig.1i pSer-302 cMyBP-C

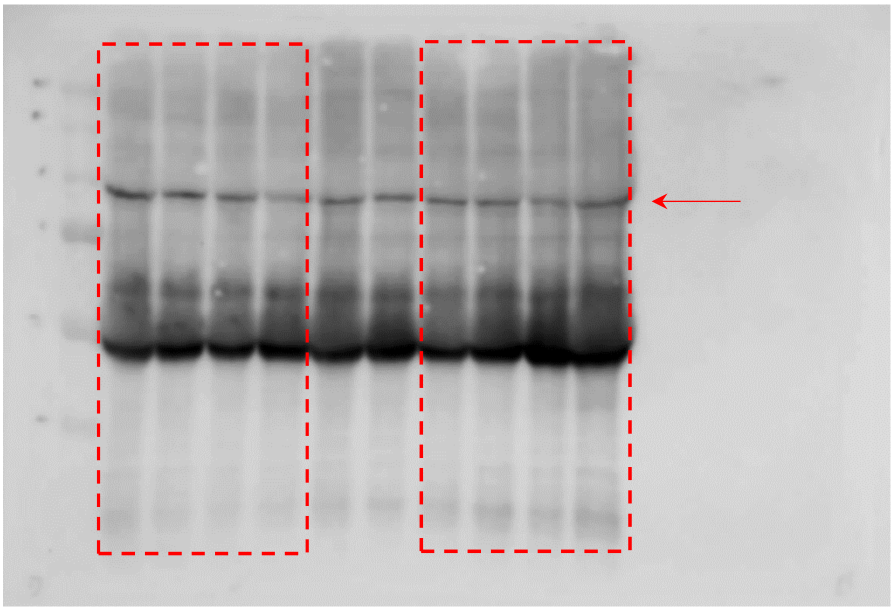

Fig.1i α-Actinin

Full Western blot membranes. Red rectangles show the selected part, red arrow shows displayed bands in the figures

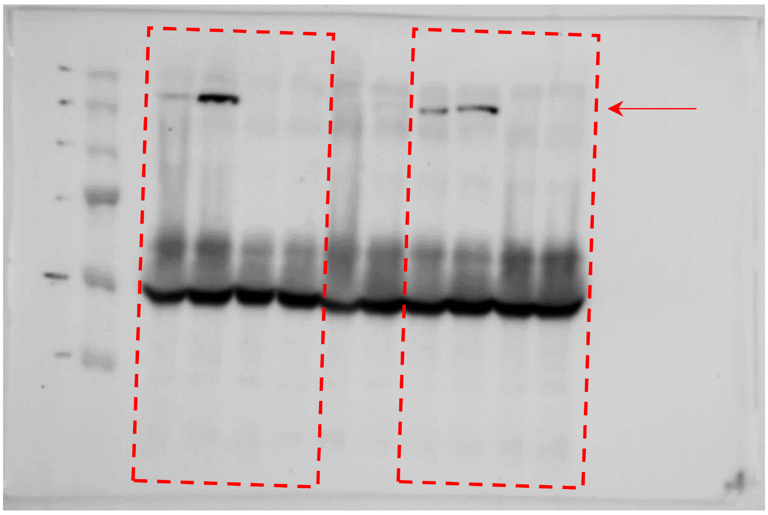

Fig.1i pSer-282 cMyBP-C

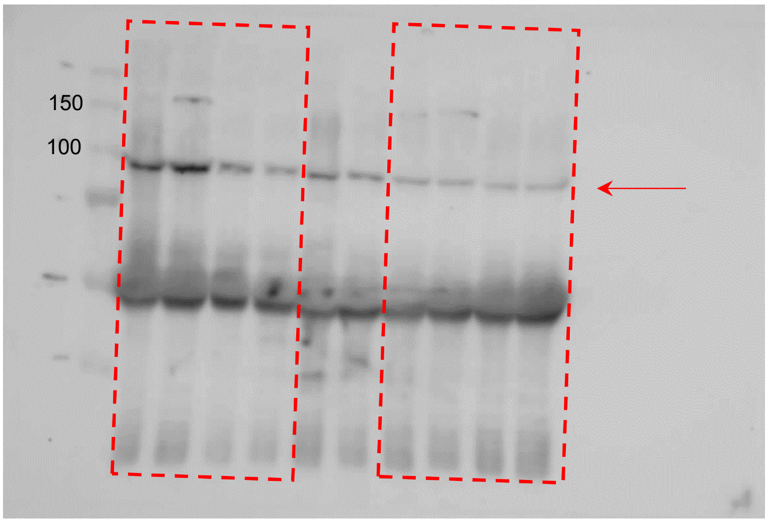

Fig.1i α-Actinin
